# Bacterial biofilm development during experimental degradation of *Melicertus kerathurus* exoskeleton in seawater

**DOI:** 10.3934/microbiol.2018.3.397

**Published:** 2018-06-07

**Authors:** Nikolina-Alexandra Xaxiri, Eleni Nikouli, Panagiotis Berillis, Konstantinos Ar. Kormas

**Affiliations:** Department of Ichthyology & Aquatic Environment, School of Agricultural Sciences, University of Thessaly, 38446 Volos, Greece

**Keywords:** bacteria, 16S rRNA, succession, chitin, marine, degradation, *Melicertus kerathurus*

## Abstract

Chitinolytic bacteria are widespread in marine and terrestrial environment, and this is rather a reflection of their principle growth substrate's ubiquity, chitin, in our planet. In this paper, we investigated the development of naturally occurring bacterial biofilms on the exoskeleton of the shrimp *Melicertus kerathurus* during its degradation in sea water. During a 12-day experiment with exoskeleton fragments in batch cultures containing only sea water as the growth medium at 18 °C in darkness, we analysed the formation and succession of biofilms by scanning electron microscopy and 16S rRNA gene diversity by next generation sequencing. Bacteria belonging to the γ- and α-Proteobacteria and Bacteroidetes showed marked (less or more than 10%) changes in their relative abundance from the beginning of the experiment. These bacterial taxa related to known chitinolytic bacteria were the *Pseudolateromonas porphyrae*, *Halomonas* *aquamarina*, *Reinekea aestuarii*, *Colwellia asteriadis* and *Vibrio crassostreae*. These bacteria could be considered as appropriate candidates for the degradation of chitinous crustacean waste from the seafood industry as they dominated in the biofilms developed on the shrimp's exoskeleton in natural sea water with no added substrates and the degradation of the shrimp exoskeleton was also evidenced.

## 1. Introduction

Marine chitinolytic, or chitinoclastic, bacteria have attracted the scientific interest several decades ago [1], primarily because this single compound is the second most abundant natural polymer after cellulose [2] and one of the major carbon and nitrogen sources for marine bacteria [3],[4]. Chitinolytic microorganisms, through the hydrolysis of glycosidic bonds, compensate the vast production of chitin in the marine environment on an annual basis, with the complete mineralization of chitin taking place in 50–140 days in surface oceanic waters [5]. The majority of chitinolytic activity takes place by free-living and animal gut associated bacteria [4],[6]. This natural cycling receives excess amounts of chitin, mostly by the accumulation of crustacean exoskeleton waste produced by commercial crustacean farming and seafood industry [7].

In 2015, 7.3 × 10^6^ tones of crustaceans were produced in marine, coastal and inland aquaculture facilities, with this production showing an increasing tendency over the years [8]. Crustacean farming byproduct, consisting mostly by heads, thorax, claws, and shells, can reach ca. 45% by shellfish weight [9]. Chitin content in this type of waste can be 15–40%. These chitinous waste, reaching up to 40% of chitin [10],[11], can cause changes in the trophic state of the aquatic environments where it is discarded, as it is prone to biological degradation by autochthonous bacteria [12] or can even impose health risks due to pathogens colonization [13],[14]. It has been proposed that the treatment-for-biodegradation of this anthropogenically produced chitinous material via the activity of efficient chitinolytic bacteria, is maybe one of the best and more ecofriendly ways to tackle this environmental issue [15]. Moreover, this biotechnological process can bring additional commercial benefits such as the production of other useful compounds, e.g. chitosan and chitooligosaccharides, that could be supplied to other industrial sectors such as food and pharmaceutics industries [16].

The biodegradation of chitinous materials requires demineralization and deproteinization, with some bacteria being able to do both [17]. Some of the known bacterial taxa involved in these steps during the biological treatment of crustacean shell wastes are *Lactobacillus* spp. *Serratia* *marcescens*, *Pseudomonas* *aeruginosa*, *P. maltophilia*, *Pediococcus acidolactici*, *Bacillus* spp. [17] are included in the rare biosphere [18] of the marine environment, where the chitinous exoskeleton wastes are produced and, thus, isolation of similar strains for subsequent inoculation is required. However, natural marine waters are most likely to contain bacterial taxa with various metabolic features that could be selected via certain experimental conditions in order to promote chitinolytic activity.

The aim of this study was to investigate (a) whether the chitin-containing exoskeleton of a commercial fisheries' shrimp species can be degraded in untreated sea water by naturally occurring bacterial communities; and (b) the diversity, succession and inferred chitinolytic potential of these bacteria. We used experimental unamended sea water batch cultures of the shrimp *Melicertus kerathurus* exoskeleton and monitored the biofilm formation on the exoskeleton by scanning electron microscopy (SEM) along with the bacterial 16S rRNA gene diversity of the biofilms. Our results depict the most likely bacterial species that could be furthered assessed for the biodegradation of chitin containing animal residuals such as shrimp shell waste.

## 2. Materials and methods

### 2.1. Experimental setup

*Melicertus kerathurus* inhabits marine and estuarine muddy sands between 0 and 90 m. Its average length is 110 to 140 mm and 130 to 170 mm for male and female individuals, respectively. This species is fished for all along the Mediterranean coasts and is an inshore fishery, because of its large size and excellent taste [19]. Freshly fished individuals of the shrimp *M. kerathurus* were acquired from the local fish market in Volos, central Greece, in February 2017 and they were transported to the laboratory in cooled foam boxes in less than one hour. In the laboratory, under aseptic conditions, the carapace was removed, cut into pieces of ca. 1 cm^2^ each and rinsed three times by gentle shaking in particle free autoclaved sea water before being added in the sea water batch cultures.

Twenty five exoskeleton carapace fragments were added to each of the three triplicated batch cultures differing only in the contained sea water: (a) seawater sterilized (coded as S) by double filtration through 0.1 µm in order to promote the growth of indigenous exoskeleton bacteria on the nutrients supplied by the sweater and the exoskeleton itself; (b) seawater filtered through 2 µm (coded as B) in order to promote the growth of natural marine bacteria—in the absence of their grazers—and indigenous exoskeleton bacteria on the nutrients supplied by the sweater and the exoskeleton itself; and (c) artificial seawater (deionized water with 3.5% NaCl, coded as A) in order to promote the growth of indigenous exoskeleton bacteria growth on nutrients originating only by the exoskeleton itself. No inorganic nutrients or organic substrates amendments took place. All batch cultures were incubated in the dark at 18 °C and under constant shaking until macroscopic signs of disintegration of the exoskeleton fragments were obvious (max. 12 days). Sampling of exoskeleton fragments was conducted at 0, 6, 9 and 12 days for scanning electron microscopy (SEM) and bacterial diversity analyses.

### 2.2. Scanning electron microscopy

For SEM, the exoskeleton samples were fixed in 2.5% glutaraldehyde in 0.1 M sodium cacodylate solution for 12 h. After fixation, samples were rinsed at both sides with 0.1 M sodium cacodylate solution and dehydration by immersion in a graded alcohol series (30%, 50%, 60%, 70%, 90%, 95% and 100%). The samples were covered with a thin layer of gold using a sputter coater (Bal-tec SCD 004), before their examination under a scanning electron microscope (Cambridge Stereoscan 240). Pictures were taken at various magnification for each sample.

### 2.3. Bacterial cell volume

For each treatment, the dimensions of 100 bacterial cells were measured every two days by the SEM photos. The long axis (D) and the small axis (d) of each bacterial cell were measured from S.E.M. pictures (Figure S1). The bacterial biovolume was calculated by the following formula:

$$Cell\;volume=3.14\times {\left(\frac{d}{2}\right)}^{2}\times D$$ (1)

### 2.4. Bacterial diversity

Each sample consisted of three individual exoskeleton fragments, one from each replicate per treatment, being pooled together immediately before the DNA extraction procedure. Bulk DNA from each of the pooled sample was acquired by using the PowerMax Soil DNA Isolation kit (MoBio, Carlsbad, CA, USA) according to manufacturer's protocol with minor modifications. The Illumina MiSeq 2 × 300 bp platform was used, targeting the V3–V4 region of the 16S rRNA gene by using the primer pair S-DBact-0341-b-S-17 (5′-CCTACGGGNGGCWGCAG-3′) and S-D-Bact-0785-a-A-21 (5′-GACTACHVGGGTATCTAATCC-3′) [20]. DNA library preparation and sequencing were performed at the facilities of MRDNA Ltd. (Shallowater, TX, USA) according to standard procedures provided by the manufacturer. All resulting data were processed with the MOTHUR software (v.1.38.0) [21]. Quality control of data analysis included flowgrams denoising by PyroNoise software [22], keeping only the sequences with ≥350 bp with no homopolymers of ≥8 bp. The remaining sequences were aligned in the SILVA 126 database [23]. The sequences were binned into operational taxonomic units (OTUs) and were clustered based on average neighbor algorithm at 97% the sequence similarity cut-off [24],[25]. The unique OTUs were taxonomically classified by using the SILVA 126 database [23]. The batch of sequences from this study can be accessed at the Short Reads Archive (http://www.ncbi.nlm.nih.gov/sra) with accession number SRP134267.

## 3. Results

### 3.1. Biofilm development

**Figure 1.**
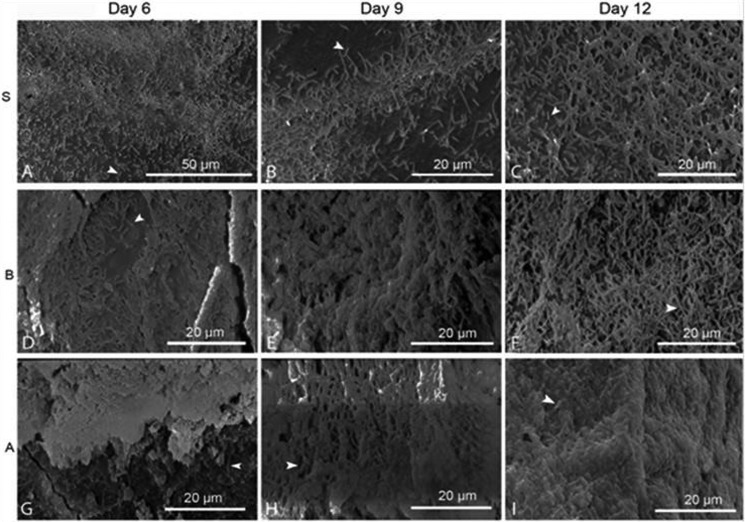
Bacterial biofilm formation on the exoskeleton of *Melicertus kerathurus* in sterile (S) (A–C), bacteria-only (B) (D–E) and artificial (A) seawater (G–I) containing treatments at days 6, 9 and 12.

**Figure 2.**
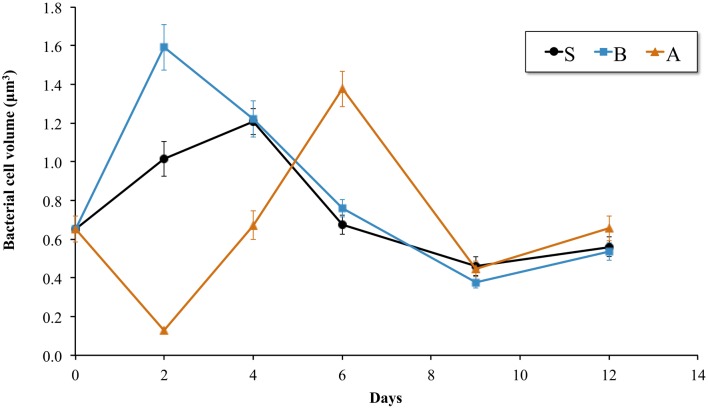
Bacterial cell biovolume of the biofilm on the exoskeleton of *Melicertus kerathurus* in the sterile (S), bacteria-only (B) and artificial (A) seawater containing treatments. Bars indicate standard error of mean N = 100 cells per sampling.

SEM observations showed that biofilm development progressed gradually already from the sixth day (Figure 1) and reached maturation, i.e. cracks in the exoskeleton fragments and detachment of the biofilm, after 12 days. The bacterial cell biovolume showed different patterns in the three treatments (Figure 2). Its highest values occurred on d4, d2 and d6 for the S, B, and A treatments, respectively, indicating different growth rates of the biofilm populations. After maximal growth, the lowest biovolume was monitored on d9 in all treatments.

### 3.2. Bacterial biofilm diversity

A total of 429 OTUs were found across all samples which belonged to 20 major taxa (phyla or subphyla; Figures S2 and S3). The most abundant taxon was the γ-Proteobacteria, with 36.8% of the total OTU number belonging to this group, followed by the Bacteroidetes (24.0%) and the α-Proteobacteria (17.0%). The rest of the phyla included ≤5.1% of the total OTU number. The number of families (Figure S3) of each major taxon was proportional to the number of OTUs in each group (R^2^ = 0.86, *p* < 0.05).

**Figure 3.**
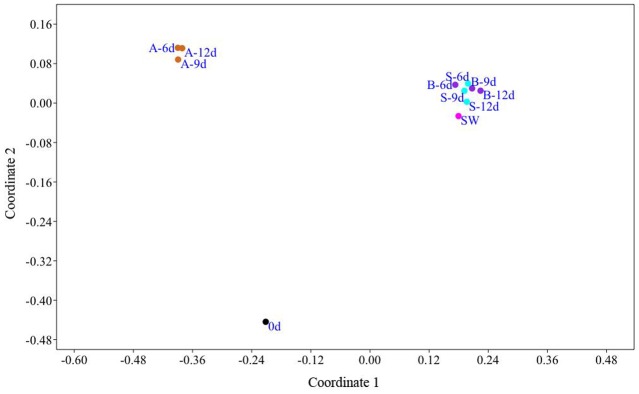
Non-metric multi-dimensional scaling (nMDS) based on Bray-Curtis similarity of the relative abundance of the biofilm bacterial operational taxonomic units (OTUs) found on the exoskeleton of *Melicertus kerathurus* in the sterile (S, blue dots), bacteria-only (B, purple dots) and artificial (A, brown dots) seawater containing treatments. Black dot (d0) represents the initial bacterial OTUs before inoculating at the different treatments; Pink dot represents natural seawater bacterial community.

Based on the OTUs abundance, non-metric multidimensional scaling separated the exoskeleton samples at d0 and the artificial water samples (Figure 3). Despite that in every sample a total of 151–285 OTUs occurred (Table 1), the number of most dominant ones (≥75% cumulative relative abundance), was lower in the exoskeleton biofilms after day 6 (3–15 OTUs) compared to the initial exoskeleton sample (23 OTUs). In each sample, the most dominant OTU had ≥16.6% relative abundance.

**Table 1.** Biofilm bacterial 16S rRNA gene diversity during experimental degradation of *Melicertus kerathurus* (Mk) exoskeleton in seawater. OTU: Operational taxonomic unit(s).

Time (days)	Treatment	Most abundant OTU	Reads	Total OTU	No. of the most dominant OTUs (cumulative relative dominance ≥ 75%)
		Dominance (%)			
		Closest relative			
	Sea water	MCB012	77,521	279	13
		20.9%			
		*Balneola alkaliphila*			
0	Mk exoskeleton	MCB003	7,783	159	23
		17.3%			
		*Pseudoalteromonas porphyrae*
6	S	MCB005	126,317	247	3
		38.7%			
		*Colwellia asteriadis*			
	B	MCB001	68,571	198	7
		37.5%			
		*Reinekea aestuarii*			
	A	MCB004	74,820	151	4
		37.2%			
		*Halomonas aquamarina*			
9	S	MCB0001	78,847	245	9
		26.7%			
		*Reinekea aestuarii*			
	B	MCB001	83,662	239	9
		31.6%			
		*Reinekea aestuarii*			
	A	MCB003	77,454	162	4
		55.8%			
		*Pseudoalteromonas porphyrae*			
12	S	MCB0005	74,228	285	15
		16.6%			
		*Colwellia asteriadis*			
	B	MCB001	87,921	264	8
		28.4%			
		*Reinekea aestuarii*			
	A	MCB004	76,925	176	6
		24.0%			
		*Halomonas aquamarina*			

In total, 10 OTUs had >10% at least in one time point and treatment. From this group, the OTUs related to *Pseudolateromonas porphyrae* and *Halomonas aquamarina* were found in all three treatments, while *Reinekea aestuarii* was found only in the S and B treatments. The rest of the OTUs, which were found only in one of the treatments were related to *Compostimonas* spp., *Pseudoalteromonas ruthenica* (in S), *Flavobacterium* spp. and *Tenacibaculum discolor* (in B) and *Vibrio crassostreae*, *Ruegeria* sp. *Alteromonas macleodii* (in A). The rest of the OTUs showed minor changes (<10%) in their relative abundances (Figure S3).

A comparable proportion of total OTUs per treatment (17.7–20.6%) was shared from d0 to d12 but the number of unique OTUs decreased from d0 to d12 (Figure S4). A similar pattern of comparable shared OTUs (42.6–45.9%) and decreasing unique OTUs was also observed between the different treatments in each sampling point (Figure S4). Regarding only the shared OTUs found in each treatment (Figure S5), 59 of them (76.6%) were also shared in all three treatments, indicating that these OTUs represent Bacteria that can be present on the *M. kerathurus* exoskeleton regardless of their origin and supplied nutrients (seawater vs. exoskeleton).

## 4. Conclusions

In this paper we describe the growth and succession of the bacterial biofilms developed during the degradation of exoskeleton fragments of the marine shrimp *Melicertus kerathurus* in unamended sea water batch cultures. We aimed at depicting which bacteria are most likely to be favoured for growth on the exoskeleton as biofilms. Only Proteobacteria (mostly γ- and α-Proteobacteria) and Bacteroidetes were favoured for abundant growth, i.e. changes in their relative abundance for ≥10% in the biofilms (sensu [26]) and these taxa are discussed further in this paper. Moreover, the bacterial cell biovolume, which is a proxy for cell growth, indicated that maximum growth occurred soon after the initiation of the experiment and reached its initial values at the end of the experiment (Figure 2), after the biofilm maturation.

The dominance of rod shaped bacteria in the biofilms (Figure 1) is in accordance with the morphology of all the dominant bacterial taxa with >10% changes found in this experiment [27]–[29]. Moreover, their ability to growth fast is also depicted by their high copy number of their 16S rRNA gene (Table S1), a parameter which is in direct positive correlation with the maximum growth rate [30]. Of all the found OTUs, the ones that could be of special interest for the degradation of the *M. kerathurus* exoskeleton, are the ones that occurred in multiple treatments and exhibited considerable growth, i.e. changes of more or less than 10% of their relative abundance compared to day 0 or the previous sampling. Most of these bacteria are known chitinolytic taxa [4],[15].

The two bacterial species which were present in all treatments and increased during the experiments for more than 10% were *Pseudolateromonas porphyrae* and *Halomonas* *aquamarina*. *P. porphyrae* growth changed more than 10% from the initial sample, only in the artificial sea water treatments, indicating its ability to outcompete other bacteria in the incubation conditions used in this experiment. The genus *Pseudoalteromonas* is widespread in the marine environment with several of its known species having chitinolytic and alginolytic properties [27]. *P. porphyrae* was first isolated from the decayed seaweed *Porphyrae* *yezoensis* [31] and it is associated with aquatic biofilms [32],[33]. The two available genomes of this species [34] contain chitinase genes which in combination with their occurrence in all of our treatments along with its high 16S gene copy number, i.e. indicative of rapid growth, suggest that it is a very potent candidate for chitin degradation of the shrimp's exoskeleton. *P. ruthenica* is among the major biofilm formers in the marine environment [35]. It has been first described from two marine molluscs, the mussel *Crenomytilus* *grayanus*, and the scallop *Patinopecten* *yessoensis*, but it has been found also in the bluefin tuna *Thunnus maccoyii* [36] and the shrimp *Litopenaeus vannamei* [37]. It is known to be able to degrade chitin [38] and has anti-bacterial properties against pathogenic bacteria [39],[40].

The growth of *Halomonas aquamarina* was also highest in the artificial sea water treatment, indicative of its marine to moderately halophilic properties (e.g. [41]–[43]) and also its ability to sustain its growth with only the exoskeleton itself as growth substrate. Its halophilic nature is also in accordance with the fact that the biofilm of *H. aquamarina* has been shown to be facilitated by higher salt concentrations in soil [44]. *H. aquamarina* is a species related to marine crustacean exoskeleton in several ways. It has been reported to contribute to the formation of bioflocs in the shrimp *Litopenaeus vannamei* growth ponds [45] and it is also considered to have beneficial properties as a probiotic for the same shrimp [46]. Moreover, its widespread occurrence in marine biofilms [47] and its ability to grow by using complex hydrocarbons has also been shown [48],[49] suggesting that it can be used for the degradation of chitinous exoskeleton.

*Reinekea aestuarii* is known to hydrolyse chitin [50] and it is also related to algal polysaccharides during phytoplankton blooms [51]. As it exhibited important growth only in the natural sea water containing treatments, it is more likely to require nutrients and substrates from the water and/or the exoskeleton itself.

*Colwellia asteriadis* grew only in the sterile sea water and this is probably due to its ability to hydrolyse chitin [52] but its biofilm formation capacity is also known for other biotic and abiotic surfaces of the marine environment [53]–[56].

One member of the biofilms developed solely in the sterile sea water treatment was related to the genus *Compostimonas*. The only described species of the genus is *Compostimonas* *suwonensis* which does not assimilate N-acetylglucosamine, the major constituent of chitin [57]. For this reason, it is likely that it does not contribute to the exoskeleton contribution. Chitin degradation by Actinobacteria has been reported only for rare members of the phylum [58].

A *Flavobacterium*-related bacterium was among the biofilm constituents in the treatments containing natural marine bacterial assemblages only. Members of the genus *Flavobacterium* are widely distributed in, mostly, aquatic habitats, both freshwater and marine [59] and are known to participate in biofilms [56],[60]. As the taxonomic assignment to a specific species of the genus is not feasible in our study, possibly due to the short length sequence, its ability to be actively involved in the exoskeleton degradation remains dubious.

Another bacterium which grew only in the sea water bacteria containing treatment was *Tenacibaculum discolor*. Members of the *Tenacibaculum* genus is related to fish disease [61],[62]. *T. discolor* has been isolated from a diseased sole (*Solea senegalensis*) and has also been found in the bluefin tuna *Thunnus maccoyii* [36]. Banning et al. [63] have reported its bacteriolytic behavior and the fact that we found it more abundant in the bacterial-containing treatment, along with the fact that in the literature it is not reported to be able to grow on chitin, most likely its growth is associated with the attachment on other bacteria of the biofilm.

*Vibrio crassostreae* grew significantly in the artificial sea water containing treatment. It has been isolated from the haemolymph of the oyster *Crassostrea gigas* [64], is a known member of marine biofilms as it has been associated with several marine surfaces such as the red coral *Corallium rubrum* [65], the invasive green alga *Caulerpa cylindracea* [66], molluscs [67] the polychaete *Myxicola infundibulum* [68] oysters [69], the mussel *Mytilus coruscus* [70] several fish [71],[72] or even microplastics [73]. However, its most interesting properties related to crustaceans exoskeletons have only recently been reported. *V. crassostreae* seems to be associated with the tail fan necrosis of spiny lobsters where is chitinolytic ability was also shown [74]. This biofilm former, as many *Vibrio* spp. have chitin binding and utilization genes [75] could be actively involved in the exoskeleton degradation of the *M. kerathurus*, acquiring its nutrients solely from the shrimp's exoskeleton and benefiting from the exclusion of other bacteria not able to grow in the artificial seawater.

The last two bacteria with significant growth in the artificial sea water containing treatments were associated with *Ruegeria* spp. and *Alteromonas macleodii*. Although the genus *Ruegeria* is associated with marine surfaces [76],[77] there is no evidence that members of this genus are hydrolyzing chitin [28]. Here, it grew at the late stage of the biofilm, possible feeding by other bacteria and their metabolites. *Alteromonas macleodii*, with a specific ecotype existing in the Mediterranean Sea [78],[79] is a ubiquitous copiotroph [80]–[84] that is favoured by increased salinity [41],[85] and is functionally selected as a keystone species with the cyanobacterium *Trichodesmium* [85],[86]. Its copiotrophic nature in combination with its high growth rate, as inferred by its multiple 16S rDNA copy number (sensu [30]) and its ability to process complex algal polysaccharides [48],[87]–[89], coral mucus [90], barnacles [91] and other refractory organic matter in the sea [92], could make it a protagonist in the chitin degradation of *M. kerathurus* exoskeleton as well. For example, a strain of *A. macleodii* has been found to have a spectacular enzyme activity for the degradation of complex organic substrates [93]. Its surface-associated lifestyle is also supported by its probiotic use in aquaculture [94] and even deep-sea hydrothermal vent shrimps [95] and polychaetes [96]. However, it might be outcompeted by others as it did not perform that well in the other two treatments where other bacteria and or substrates from the surrounding environment existed.

In conclusion, our study extends the list of bacterial with the potential to degrade crustacean exoskeletons as biofilms in natural sea water. We found that exoskeleton biofilm development takes place in just a few days with some of the bacteria being selected for rapid growth in these biofilms originating either from the marine environment or from the natural microbiota of the exoskeleton itself. An indirect indication of chitin degradation by the biofilm came from the fact that pH during the experiment was reduced from 8.1 at the beginning to 6.7 at the end of the experiment. Activity of chitinase genes is favoured in lower pH values than those prevailing in natural sea water [6],[17],[97]. Future research is required to assess whether these bacterial species could be appropriate degraders for the management of crustacean cell waste in an ecofriendly way as they can grow in natural sea water.
