# Supplementary material for: Bacterial biofilm development during experimental degradation of Melicertus kerathurus exoskeleton in seawater
Source: AIMS Microbiol. 2018 Jun 7;4(3):397–412. doi: 10.3934/microbiol.2018.3.397 (PMC6604942; doi:10.3934/microbiol.2018.3.397)
Supplement: Supplementary file 1 [file microbiol-04-03-397-s1.pdf]

Microbiology

AIMS Microbiology, 4(3): 397–412.
DOI: 10.3934/microbiol.2018.3.397
Received: 14 April 2018
Accepted: 29 May 2018
Published: 07 June 2018

http://www.aimspress.com/journal/microbiology

Research article

Bacterial biofilm development during experimental degradation of *Melicertus kerathurus* exoskeleton in seawater

Nikolina-Alexandra Xaxiri, Eleni Nikouli, Panagiotis Berillis and Konstantinos Ar. Kormas*

Department of Ichthyology & Aquatic Environment, School of Agricultural Sciences, University of Thessaly, 38446 Volos, Greece

* **Correspondence:** Email: kkormas@uth.gr, kkormas@gmail.com; Tel: +302421093082; Fax: +302421093157.

Supplementary

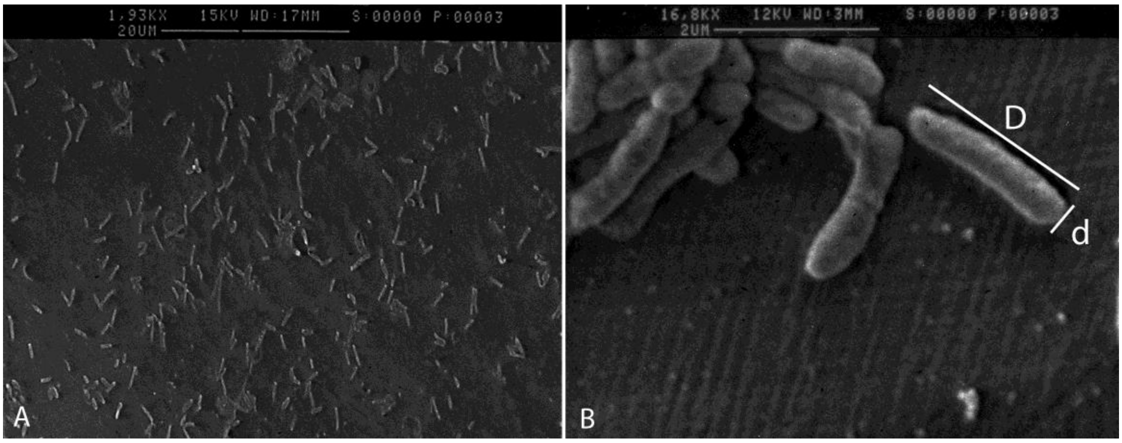

Figure S1. (A): Attached bacteria on the exoskeleton of *Melicertus kerathurus* at 0 days; (B): measurements of the long; (D) and small; (d) axis of the bacterial cells.

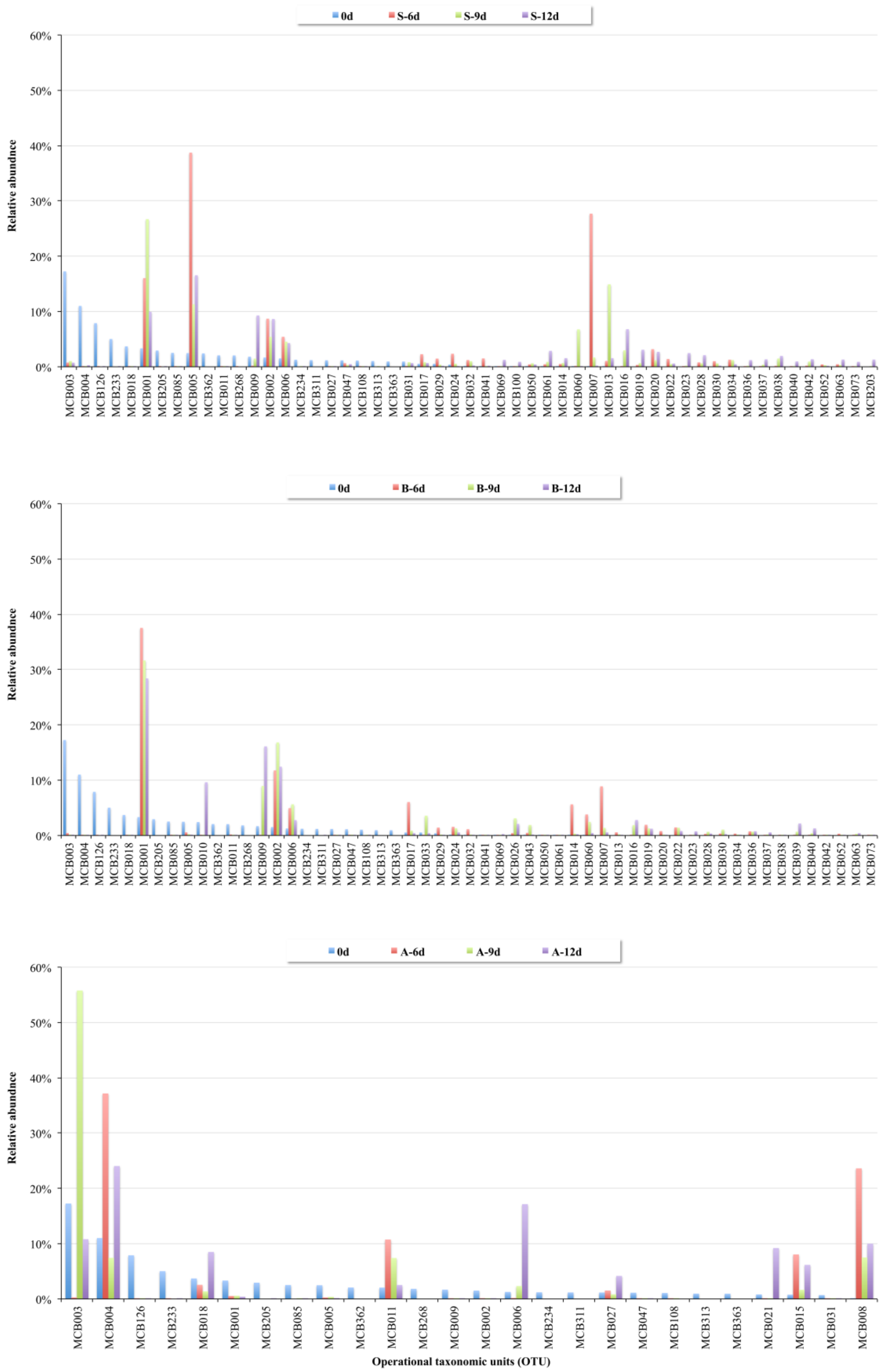

Figure S2. Operational taxonomic units with up to 75% cumulative relative abundance in the sterile (S), bacteria-only (B) and artificial (A) seawater containing treatments. d: Days.

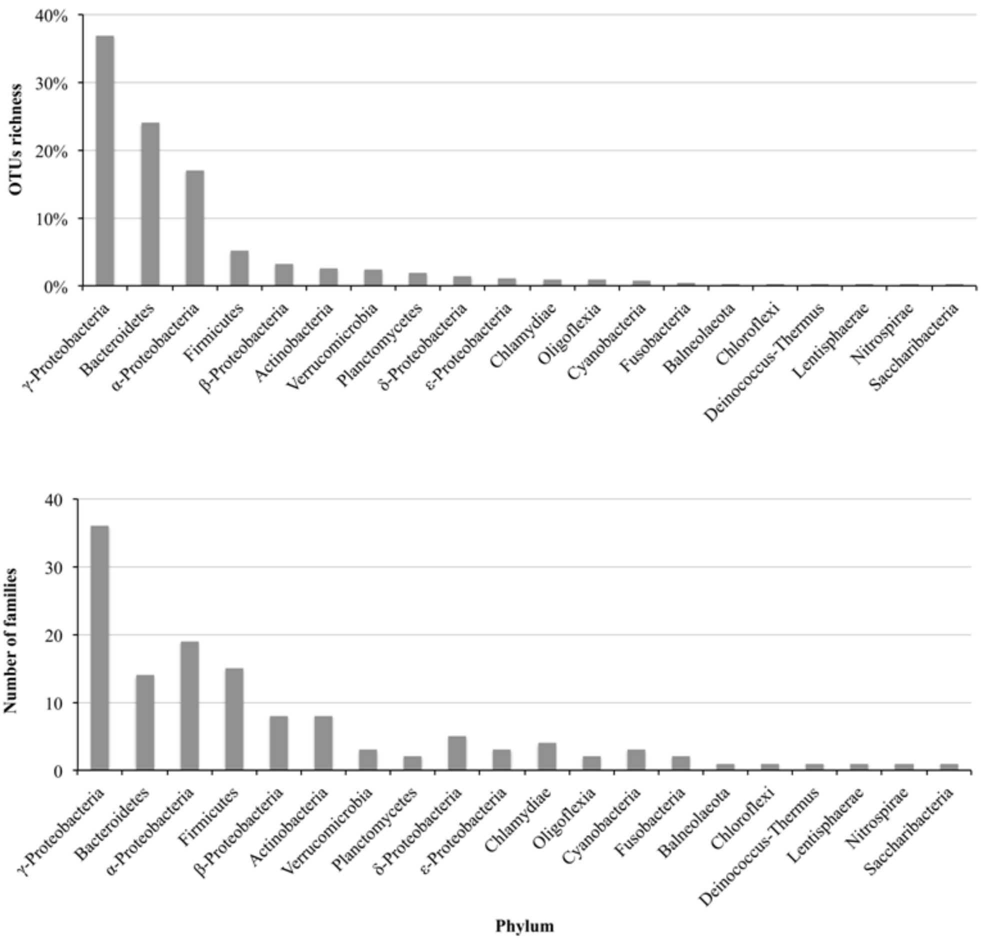

Figure S3. Higher bacterial taxa of the operational taxonomic units found in the sterile (S), bacteria-only (B) and artificial (A) seawater containing treatments.

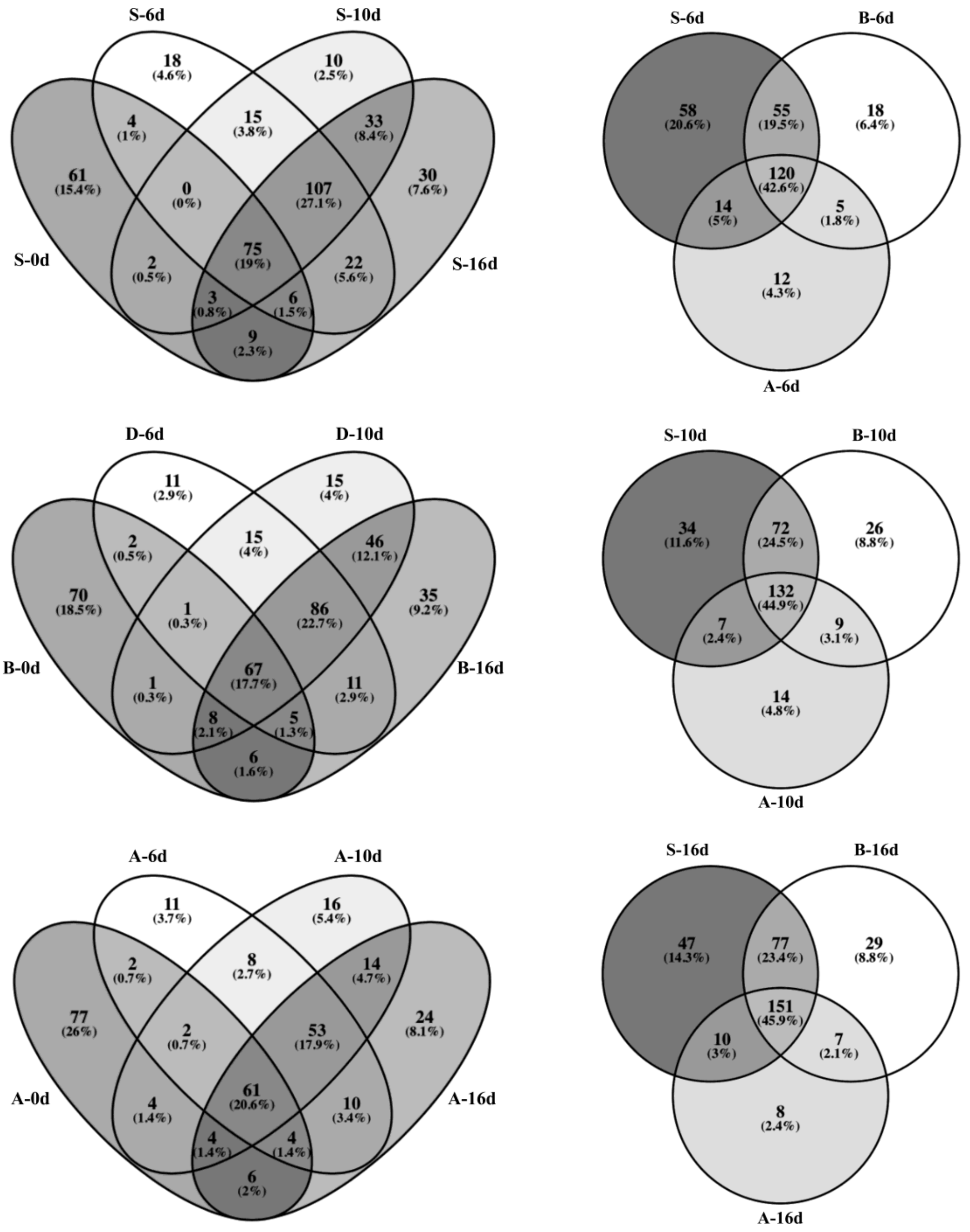

Figure S4. Venn diagrams of the shared and unique operational taxonomic units per treatment (left panel) and per day (right panel). S: sterile; B: bacteria-only; and A: artificial seawater containing treatments. d: Days.

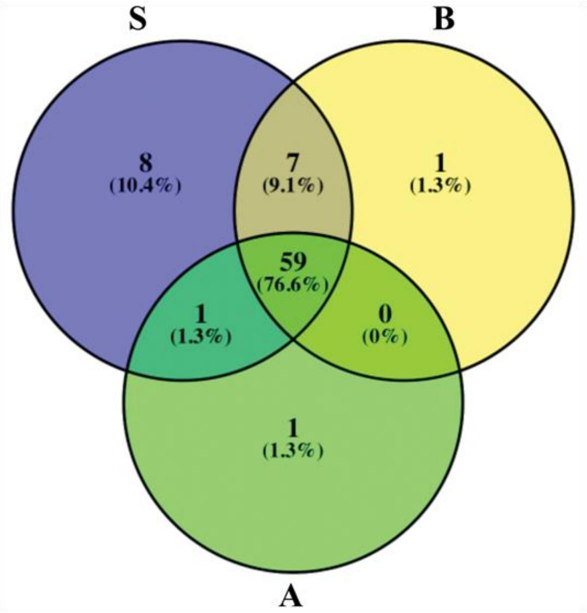

Figure S5. Venn diagram of all the operational taxonomic units shared among treatments in the sterile (S), bacteria-only (B) and artificial (A) seawater containing treatments. d: Days.

Table S1. 16S rRNA gene copy number from the rrnDB* (https://rrndb.umms.med.umich.edu) and Microbial Genomes (https://www.ncbi.nlm.nih.gov/genome/microbes/). Accessed on 01 March 2018.

Taxon	No. of genomes	Average no. of 16S rDNA copies	Standard deviation
Alteromonas macleodii	6	5	0
Colwellia spp.	6	7	1.4
Halomonas spp.	10	4.7	1.6
Pseudoalteromonas spp.	24	8.8	1.6
Reinekea spp.	1	5	-
Ruegeria spp.	3	4	0.8
Vibrio spp.	122	10.1	1.9

*Stoddard S.F, Smith B.J., Hein R., Roller B.R.K. and Schmidt T.M. (2015) *rrn*DB: Improved tools for interpreting rRNA gene abundance in bacteria and archaea and a new foundation for future development. *Nucleic Acids Research* 2014; doi: 10.1093/nar/gku1201.

AIMS Press

© 2018 the Author(s), licensee AIMS Press. This is an open access article distributed under the terms of the Creative Commons Attribution License (http://creativecommons.org/licenses/by/4.0)
